# Isolation and Identification of Pathogens Causing Blue Mold of Lanzhou Lily during Postharvest Storage and Control of Disease and Mycotoxin Accumulation by Ozone Treatment

**DOI:** 10.3390/jof9111091

**Published:** 2023-11-08

**Authors:** Hui Zhang, Jihui Xi, Zhiguang Liu, Minxuan Chen, Zhenhang Lu, Huali Xue, Yang Bi

**Affiliations:** 1 College of Science, Gansu Agricultural University, Lanzhou 730070, China; gsnydxzh@163.com (H.Z.);; 2 College of Food Science and Engineering, Gansu Agricultural University, Lanzhou 730070, China

**Keywords:** Lanzhou lily, postharvest disease, morphological identification, molecular biology technique identification, mycotoxin, ozone

## Abstract

Blue mold (*penicilliosis*) is a common disease of Lanzhou lily (*Lilium davidii* var. *willmottiae*) during postharvest storage, which not only seriously affects the appearance and reduces the quality of lily bulbs, but also leads to the accumulation of mycotoxins in rotten lily tissues, seriously endangering human health. Therefore, it is of great significance to clarify the main isolates causing postharvest blue mold of fresh Lanzhou lily and put forward effective measures to control the disease caused by these pathogens. In this study, pathogens were isolated and purified from the naturally diseased blue-mold tissue of Lanzhou lily, and then morphological and molecular biology techniques were applied to identify the isolates, verify the pathogenicity, determine the disease index and disease incidence, and finally, to analyze the control effect of ozone treatment on the blue mold of lily scale and mycotoxin accumulation. The results indicated that the main isolates causing postharvest blue mold of lily were *Talaromyces adpressus*, *Penicillium gladioli*, *T. calidominioluteus,* and *P. polonicum*. The pathogenicity test showed that *P. gladioli* and *P. polonicum* had a higher disease index than *T. calidominioluteus* and *T. adpressus*. Ozone treatment significantly reduced the incidence of disease caused by *P. gladioli* and *P. polonicum*, and effectively controlled the accumulation of patulin. This study characterized the main pathogens causing blue mold of postharvest Lanzhou lily during storage, and confirmed ozone application has a significant inhibitory effect on blue mold development and patulin accumulation in Lanzhou lily, which could be helpful in commercially controlling blue mold of postharvest Lanzhou lily during storage.

## 1. Introduction

Lanzhou lily is the only famous edible sweet lily in China [1]. As a special product of Lanzhou in Gansu province, Lanzhou lily has a high nutritional value and can be used as food and as medicine [2,3]. Lanzhou lily bulbs are white and yield large slices, with a sweet and crisp taste. Lanzhou lily is one of the most famous cash crops in Lanzhou. Fresh Lanzhou lily generally requires 6–12 months of storage after harvest. However, during postharvest storage, it is highly susceptible to infection and decay by pathogenic microorganisms. This causes great economic loss to lily growers and processors [4]. Blue mold is a common disease during postharvest storage of Lanzhou lily, with disease symptoms mainly characterized by bulb lesions, with a dark-brown and bluish-green mold layer on the top, gradual decaying of internal scales, and finally, dry rot of the bulb. It usually takes 2–3 months for the bulbs to decay completely. In particular, if the bulb tissue is injured during harvesting or transport, or there is high temperature, high humidity, or poor ventilation in the storehouse, the disease will be more serious [4]. Ju’s research group [5] suggested that the main pathogens causing scale decay of Lanzhou lily in the process of aeroculture are *Fusarium oxysporum* and *F. Fuji*, with a disease incidence of 95.00% and 75.00%, respectively. Wang et al. [6] found that gray mold caused by *Botrytis cinerea* was the most important and common leaf disease in the field [7,8], and that blue mold caused by *Penicillium* spp. occurred mainly during the storage period of lily bulbs, with brown indentations on the infected scales or at the base of the scales [9]. At present, low-temperature storage is the most widely used strategy to control disease in Lanzhou lily [10]; however, it is well known that some pathogens still grow even under low-temperature conditions [11], which not only seriously reduces the edible value of Lanzhou lily, but also brings huge risks for human health. Chemical synthetic fungicides are also used to control disease in fresh Lanzhou lily [12]; nevertheless, these fungicides have a series of problems, such as drug residues, environmental pollution, and drug resistance. Therefore, it is particularly important to develop feasible measures to control postharvest disease of fresh Lanzhou lily. 

Ozone, as a strong oxidizing agent, has significant advantages, such as no residue, and extension of shelf life of fresh fruits and vegetables, and it has been widely applied in various fruits and vegetables [13,14]. Under the appropriate treatment concentration, ozone will not adversely affect the appearance, texture, or nutritional quality of fruits and vegetables, and can effectively extend their storage period [7,15]. Ali et al. [16] indicated that ozone treatment improved the antioxidant ability involved in defense mechanisms, thereby better controlling papaya fruit decay. Lv et al. [17] and Xi et al. [18] suggested that ozone gas fumigation significantly controlled disease occurrence and mycotoxin accumulation in fresh *Codonopsis pilosula* and *Angelica sinensis* during postharvest storage. Ozone gas has a good control effect in the preservation of fruits and vegetables and fresh Chinese medicinal materials [19]; however, whether ozone can also control the postharvest disease of Lanzhou lily and inhibit the accumulation of mycotoxins in disease tissues has not been reported.

In this study, we isolated the isolates causing blue mold of Lanzhou lily. Then these isolates were identified by morphology and molecular biology, the pathogenicity was verified according to Koch’s rule [20], the disease index and disease incidence were determined, and finally, we analyzed the control effect of ozone treatment on the blue mold and mycotoxin accumulation in lily scale caused by *P. gladioli* and *P. polonicum*.

## 2. Materials and Methods

### 2.1. Materials

Lanzhou lily was purchased from a local planting base in Qilihe District, Lanzhou, Gansu Province, without any treatment after purchase, and was directly stored at room temperature (25 °C, 30% RH) in the laboratory and refrigerated (4 °C, 80–85% RH) for 27 days for natural disease. 

Lily bulbs with healthy inner layers, and of the same size and similar thickness, were selected for pathogenic verification and subsequent ozone treatment.

### 2.2. Methods

#### 2.2.1. Isolation and Purification of Isolates

Referring to the method of Xi [13], the tissue (5 mm × 5 mm) at the junction site of diseased and healthy lily bulb was cut, and soaked in 75% alcohol for 5 s for disinfection, rinsed with sterile water 3 times to remove alcohol residue, then dried naturally at room temperature. The sterilized lily tissue was inoculated onto PDA medium and cultured at 28 °C. Colonies growing with different morphologies were separated by streak on new PDA medium and this was repeated 2–3 times until a single colony was obtained. The experiment was repeated three times; one repeat included 5 colony cultures. 

#### 2.2.2. Morphological Identification of Isolates

Colony morphology was observed on the PDA medium, and mainly included colony shape, color, and texture; colony edge; and conidia and spore morphology. For morphological identification of isolates, we referred to the method used by Navalkar and Watrud [21,22] to determine *P. spinulosum* colony morphology. For observation results, we referred to the *Fungal Identification Manual* [23]. 

Spore morphology was observed by scanning electron microscope (ULTRAPLUS, ZEISS, Oberkochen, Germany), and spores were cultured using an improved glucose content of PDA. 

Spore pedicle morphology was observed under an optical microscope (CX21FS1C, OLYMPUS, Tokyo, Japan). The spore suspension (1 × 10^6^ spores/mL) was prepared, inoculated on PDA medium, and cultured by the solid-insert method.

#### 2.2.3. Molecular Biological Identification of Isolates

DNA extraction of isolate mycelia was carried out according to the method of Lv [17]. Mycelia of fungi cultured on PDA medium were collected, ground into powder in liquid nitrogen, and lysed into platelet lysate. A phenol–chloroform mixture was then added, mixed thoroughly, and centrifuged. The water phase was then added to 1 mL ethanol solution and mixed upside-down. It was then centrifuged again without flocculation, the supernatant was discarded, and the mixture left for precipitation. Ethanol solution was added for centrifugation, the supernatant was discarded completely, the precipitate was kept, and 50–100 μL of solution was added until the precipitate was completely dissolved. Then, 2–5 μL of running glue (1% agarose) was taken, according to the electrophoretic map, and an appropriate amount of DNA solution was taken for subsequent PCR. 

The PCR amplification reaction system (50 μL) consisted of a 46–47 μL 1 × Taq PCR mixture containing 1 μL upstream and downstream primers and 1 μL template DNA. The primers (*Internal Transcribed Spacer*, *ITS*; *Btub A/B*, and *Bt*) and the sequences used are shown in Table 1. Using the extracted isolate DNA as a template, the primers were designed according to the method of Xi [18]. The PCR amplification procedure followed and consisted of pre-denaturation at 95 °C for 5 min; denaturation at 95 °C for 10 s; then, annealing at 53 °C for 10 s, extending at 72 °C for 30 s, for 35 cycles, and finally, holding at 72 °C for 5 min. The amplified product was isolated by electrophoresis on 1% agarose gel. 

The amplified fragments were sequenced by Beijing Pomerander Biotechnology Co., Ltd., Beijing, China. The sequencing results were analyzed by BLAST in the GenBank database, suitable sequences were selected, and the phylogenetic tree was constructed by the MEGA 7.0.26 (7170509-x86_64) software adjacency method. Combined with the results of molecular biology identification and morphological identification, the pathogenic fungi species were finally determined.

#### 2.2.4. Pathogenicity Testing of Isolates

Healthy bulb tissue of Lanzhou lily was soaked in 0.1% sodium hypochlorite solution for 15 min, washed with sterile water three times, and then air dried at room temperature. A pipetting gun was used to make holes on lily scales and the prepared spore suspension of 1 × 10^6^ spores/mL was inoculated into the punctured holes by spraying; the punctured but not inoculated scales were used as the control. Lily scales were stored in a plastic bag with wet filter paper to provide moisture. The bags were stored at room temperature for 15 days. Samples were taken every 5 days to calculate the disease incidence and disease index. Four groups of treatments were inoculated with four major pathogenic strains, and each treatment was repeated three times (one repeat included 10 pieces of healthy lily). After 15 days of incubation, the isolates were isolated and purified from the moldy tissue and compared with the inoculated pathogens.

The disease incidence and disease index were calculated as follows: 

Disease index = ∑ (number of disease grade × representative value of each disease grade)/(total number of plant × representative value of highest disease grade) × 100%

Disease incidence = number of diseased plants/total number of plants × 100%

For the two formulae, disease level was classified into five categories: 0, 1, 2, 3, and 4 (Table 2).

#### 2.2.5. Control of Blue Mold of Lanzhou Lily by Ozone Treatment

Based on the above results of pathogenicity verification, *P. gladioli* and *P. polonicum* were considered to be the main strains causing blue mold in Lanzhou lily. Therefore, in the next part of the study, we mainly analyzed the effects of ozone treatment on the two strains causing blue mold in Lanzhou lily. Inoculation was carried out according to Section 2.2.4. Four treatment groups were included. Ozone treatment and control group were inoculated with *P. gladioli*, ozone treatment and control group were inoculated with *P. polonicum*. Each treatment was repeated three times (one repeat included 10 pieces of healthy lily). 

Ozone gas preparation and adjustment of the ozone concentration was according to the method of Xi [18], the concentration was adjusted to 2 mg/L; the ozone entry time was 2 h, once a day; and treatment was continuous over a period of 15 d. The disease index and disease incidence were measured every 5 days using the same method as in Section 2.2.4. Lily samples were collected and ground into powder with liquid nitrogen, then kept at −80 °C for patulin assay.

#### 2.2.6. Determination of Patulin

The extraction and determination of patulin was according to the method of Zhu [24]. First, 2.0 g of the lily powder was weighed and transferred to a centrifuge tube. Then, 40 mL ethyl acetate was added to the centrifuge tube and the mixture was centrifuged at 1187× *g*, at 4 °C for 3 min. Centrifugation was repeated three times. The extract was placed into a flask, evaporated to near-dry at 40 °C, and then blown dry with nitrogen stream. Samples were redissolved with 3 mL AABS buffer and filtered using a 0.22 μm organic microporous filter membrane. The samples were then detected by high-performance liquid chromatography. 

Chromatographic conditions: C_18_ reversed phase column (250 nm × 4.6 nm × 5 μm), 276 nm wavelength, UV detector; acetonitrile: water (1:9, *v*:*v*) was used as mobile phase. Column temperature: 35 °C; sample size: 20 μL; flow rate: 0.75 mL/min.

#### 2.2.7. Statistical Analysis

Data (average value and standard deviation) were calculated using Excel 2019, and SPSS 21.0 was adopted to analyze the difference significance (*p <* 0.05). All the figures in this study were created with Origin 2021 (Northampton, MA, USA).

## 3. Results

### 3.1. Development of Lanzhou Lily during Storage

**Figure 1:**
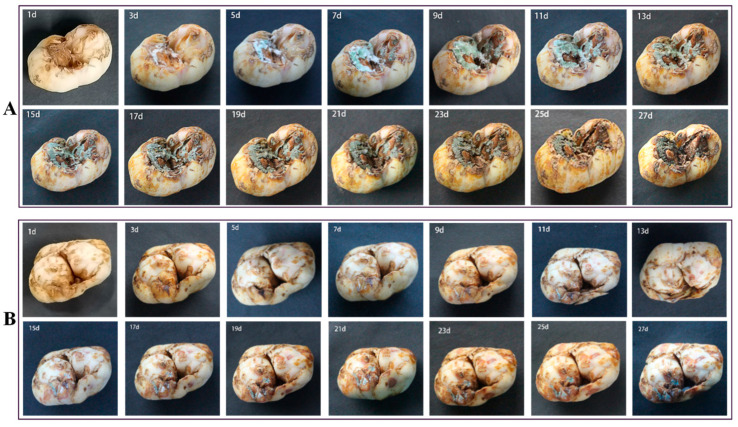
Symptoms of natural disease of Lanzhou lily after different storage periods. (**A**) Room-temperature storage and (**B**) cold-temperature storage.

It can be seen from Figure 1 that a white mold layer was found on the bulb at the 3rd day of storage, and, with the extension of storage, a green mold layer was observed. Under room-temperature storage conditions, the mold area on Lanzhou lily increased gradually from the 3rd day of storage to the 11th day. After 13 d of storage, the area of blue mold infection did not expand. At 19 d of storage, the lesion began to dry rot and the mildew layer turned dark green. Under low-temperature storage conditions, the disease symptoms appeared later than that under room-temperature conditions; the disease symptoms began on the 7th day, with a slight white moldy layer. Blue mold was observed on scales after 15 d of storage. The area of the moldy layer area gradually increased at 27 d of storage. On the whole, the disease development in bulbs stored at room temperature was faster and more serious than in those stored at low temperature.

### 3.2. Morphological Identification of Isolates

Four strains of isolates were isolated and purified from the blue mold of lily during storage, and morphology, including colony morphology, spore morphology, and spore stalk morphology, was observed. 

**Figure 2:**
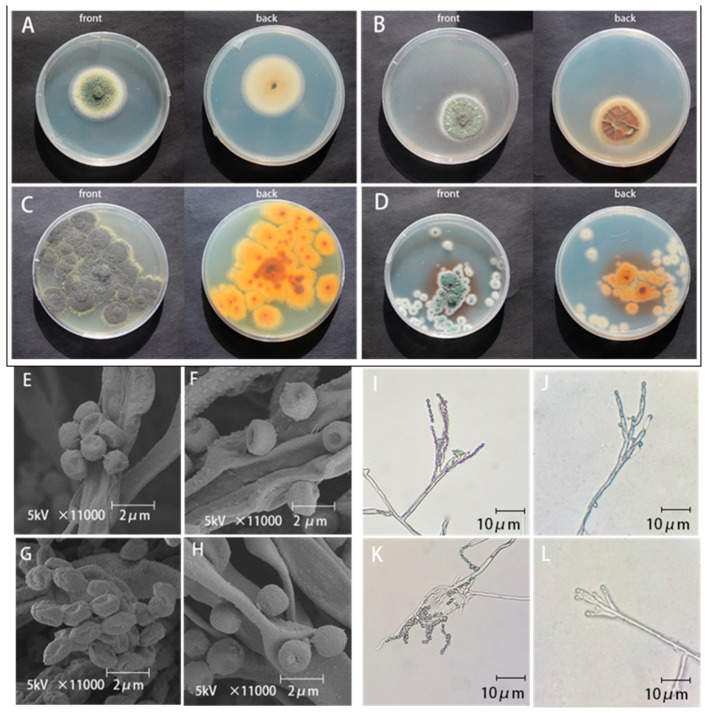
Morphological observation of isolates. (**A**) *zh-1* colony morphology, (**B**) *zh-2* colony morphology, (**C**) *zh-3* colony morphology, (**D**) *zh-4* colony morphology, (**E**) *zh-1* spore morphology, (**F**) *zh-2* spore morphology, (**G**) *zh-3* spore morphology, (**H**) *zh-4* spore morphology, (**I**) *zh-1* sporophyte morphology, (**J**) *zh-2* sporophyte morphology, (**K**) *zh-3* sporophyte morphology, and (**L**) *zh-4* sporophyte morphology.

The colony morphology of *zh-1* on PDA medium is shown in Figure 2A. The mycelium of the front colony was yellow-green, with slight variegated flesh color. It was a thin colony with no radial groove. The back of the colony was smooth. The center of the colony had a sparsely covered mycelium in a fluffy shape. Colony edges were neat and smooth. At the later stages of growth, there was a woolly and sparse mycelium covered by a cirrus mycelium. Microscopic observations of the conidia and spore morphology are shown in Figure 2E,I. The conidia were oval with smooth to slightly rough walls, and formed an irregular short chain. According to the morphological characteristics, *zh-1* was preliminarily identified as *Talaromyces adpressus*. 

The colony morphology of *zh-2* on PDA medium is shown in Figure 2B. The mycelium of the front colony was green, the colony of the isolate was raised on the plate, and the colony was thick. The back colony was brown and milky white with folds. The mycelium on the surface of the colony was dense, and the tissue shape was villous. Colony edges were neat and smooth. Microscopic observations of the conidia and spore morphology are shown in Figure 2F,J. Conidia were spherical with concave middle, smooth walls, and long peduncles. According to the morphological characteristics, *zh-2* was preliminarily identified as *P gladioli*. 

The colony morphology of *zh-3* on PDA medium is shown in Figure 2C. Conidia of the front colony were grayish green to dark green, and the mycelium was white to pale yellow. The back of the colony was smooth, unwrinkled, and brown and dark yellow. The whole margin of the colony was protruding. Microscopic observations of the conidia and spore morphology are shown in Figure 2G,K. The conidia were oval in shape and tightly chained. Based on morphological characteristics, *zh-3* was preliminarily identified as *T. calidominioluteus*. 

The colony morphology of *zh-4* on PDA medium is shown in Figure 2D. The front colony was velvet, olive green, and protruding. The dorsal mycelium of the colony was white, and the dorsal surface was smooth and dark yellowish-brown. Colony edges were blurred. Microscopic observations of the conidia and spore morphology are shown in Figure 2H,L. The spore peduncles were long and the conidia were more spherical. Based on morphological characteristics, *zh-4* was preliminarily identified as *P. polonicum*.

### 3.3. Molecular Biological Identification of Isolates

**Figure 3:**
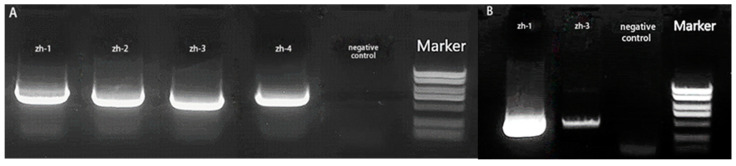
Gel electrophoresis image of PCR amplified products. (**A**) *ITS* and (**B**) *Bt*.

Four isolates were amplified by PCR using *ITS* primers, and *zh-1* and *zh-3* were further amplified by *Bt* gene primers. The amplified products were analyzed by 1% agarose gel electrophoresis, and the resulting gel electrophoresis images are shown in Figure 3A,B. The sequence sizes of four isolates amplified by *ITS* primer were 529 bp, 530 bp, 541 bp, and 530 bp, and the sequence sizes of the two pathogens amplified by *Bt* primer were 429 bp and 428 bp.

**Figure 4:**
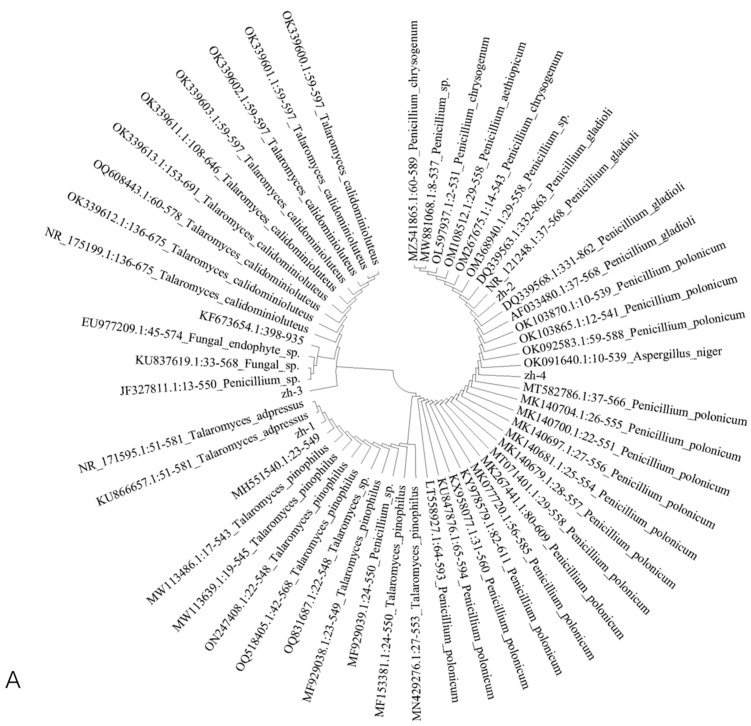

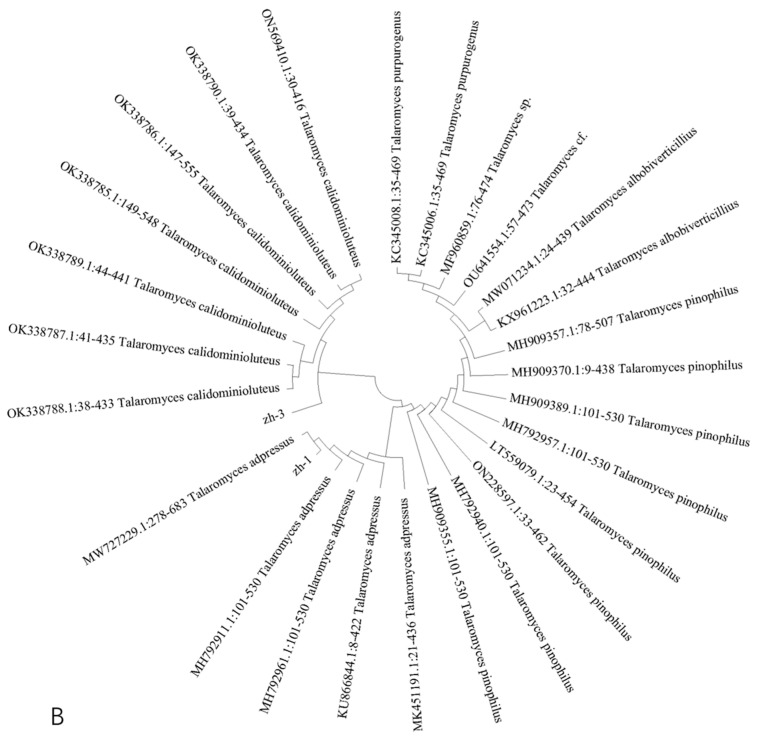
Phylogenetic trees based on genetic analysis of different strains. (**A**) *ITS* and (**B**) *Bt*.

The amplified sequences were sequenced and BLAST-compared at NCBI (https://www.ncbi.nlm.nih.gov/ (accessed on 27 April 2023)), and highly homologous sequences (more than 95.00%) with isolated strains were selected. MEGA 7.0.26(7170509-x86_64) software was used to construct the *ITS* and *Bt* phylogenetic trees based on the sequence-by-adjacency method (Figure 4).

The results showed that *zh-1* and *T. adpressus* (*KU866657.1:51-581*) were located on the same evolutionary branch of the *ITS* phylogenetic tree, with a high homology of 99.44%. *zh-3* was highly homologous to *T. calidominioluteus* (*NR_1751991.1:136-675*), and *zh-1* and *zh-3* converged on the same evolutionary branch of the *ITS* phylogenetic tree. In the *Bt* phylogenetic tree, *zh-1* had 98.03% homology with *T. adpressus* (*MW727229.1:278-683*); *zh-3* and *T. calidominioluteus* (*OK338785.1:149-548*) were located in the same branch, with a homology of 95.76%; and *zh-1* and *zh-3* were located in different branch. Combining the above analysis and morphological identification, the isolate of *zh-1* was identified as *T. adpressus*, and the isolate of *zh-3* as *T. calidominioluteus*. 

*zh-2* and *P. gladioli* (*NR_121248.1:37-568*) were located on the same evolutionary branch of the *ITS* phylogenetic tree, with a homology of 99.62%, and *zh-4* shared 100.00% homology with *P. polonicum* (*MT582786.1:37-566*) on the same cladistic branch in the *ITS* phylogenetic tree. Therefore, combined with morphological observation and the phylogenetic tree analysis, *zh-2* was identified as *P. gladioli* and the *zh-4* pathogen as *P. polonicum*.

### 3.4. Pathogenicity Verification

**Figure 5:**
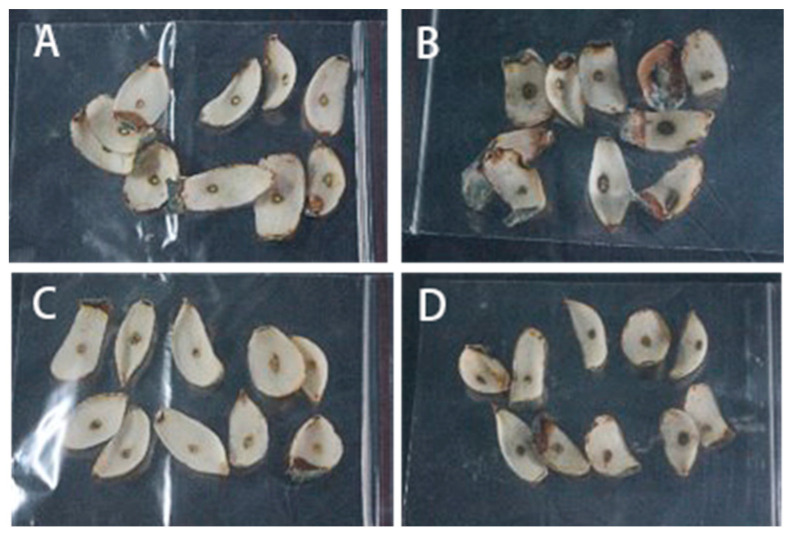
Pathogenicity testing of isolates causing blue mold of Lanzhou lily. (**A**) *zh-1*, (**B**) *zh-2*, (**C**) *zh-3*, and (**D**) *zh-4*.

**Figure 6:**
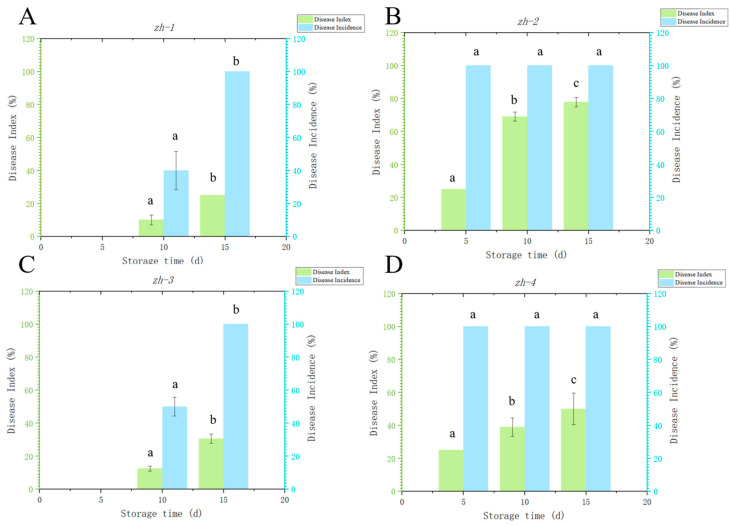
Disease incidence and disease index of *zh-1* (**A**), *zh-2* (**B**), *zh-3* (**C**), and *zh-4* (**D**). The vertical lines represent the standard error (±SE), and there were significant differences in different alphabetic representations of the variable (*p <* 0.05).

Four isolates were isolated and purified from natural disease of Lanzhou lily scales during storage by the tissue-separation method. The tieback experiment of fresh lily was carried out according to Koch’s rule. The results showed that the lily bulbs inoculated with the four isolates all showed typical disease symptoms (Figure 5). The junction tissues of health and disease of the infected lily were collected, isolated, and purified, and the isolates were re-obtained, and compared with the isolates initially isolated from the blue-mold tissue of the lily. The results showed that the re-isolated isolates had the same morphological characteristics as the original isolates, indicating that these four isolates were the main pathogens causing blue mold of Lanzhou lily. Through the analysis of the pathogenicity of these four isolates on Lanzhou lily, the results showed that the disease incidence of the four isolates was 100.00%, and the disease indices of *zh-1*, *zh-2*, *zh-3,* and *zh-4* were 25.00%, 77.78%, 30.55%, and 50.00%, respectively, after 15 d of storage (Figure 6). *zh-2* had the highest disease index, followed by *zh-4*. Therefore, *zh-2* and *zh-4* were considered as the main isolates causing blue mold of lily during storage.

### 3.5. Ozone Treatment for Disease Control

**Figure 7:**
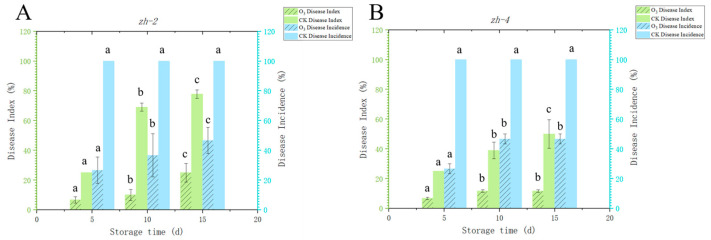
Effect of ozone treatment on the disease index and disease incidence of blue mold in Lanzhou lily inoculated with *P. gladioli* and *P. polonicum. P. gladioli* (*zh-2*) (**A**) and *P. polonicum (zh-4*) (**B**). The vertical lines represent the standard error (±SE), and there were significant differences in different alphabetic representations of the variable (*p <* 0.05).

The pathogenicity analysis of the above four isolates indicated that *P. gladioli* and *P. polonicum* were more pathogenic to Lanzhou lily. Therefore, the subsequent analysis of ozone treatment on the control effect of blue mold and mycotoxin accumulation in Lanzhou lily mainly focused on the two *Penicillium* spp. The results suggested that ozone treatment had a significant inhibitory effect on blue mold of lily. For instance, ozone treatment reduced the disease incidence and the disease index to 46.67% and 25.00%, respectively, in lily bulbs infected by *P. gladioli* after 15 d of storage (Figure 7A). Similarly, ozone treatment also decreased the disease incidence and disease index from 46.67% to 11.67% and from 100% to 50.00%, respectively, in lily bulbs inoculated with *P. polonicum* after 15 days storage (Figure 7B).

### 3.6. Effect of Ozone Treatment on Patulin Accumulation in the Lesion Tissue of Lanzhou Lily

**Figure 8:**
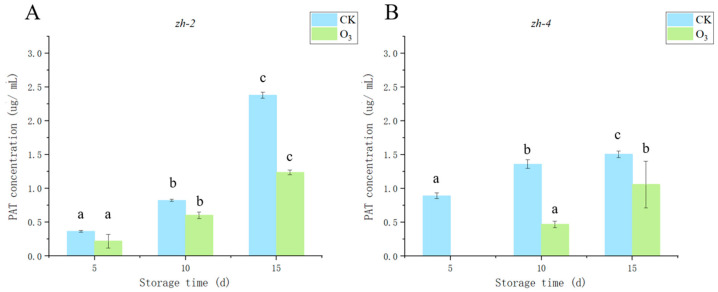
Effect of ozone treatment on patulin accumulation in Lanzhou lily inoculated with *P. gladioli* and *P. polonicum*. *P. gladioli* (*zh-2*) (**A**) and *P. polonicum (zh-4*) (**B**). The vertical lines represent the standard error (±SE), and there were significant differences in different alphabetic representations of the variable (*p <* 0.05).

*P. gladioli* (*zh-2*) infection led to the accumulation of PAT in blue mold of lily, and with the increase in storage time, the content of PAT in lily scale tissue continued to increase; nevertheless, the trend of increase in the control group was significantly (*p* < 0.05) greater than in the ozone-treated group, and the longer the ozone time treatment, the more obvious the inhibition effect observed. For instance, on the 15th day after inoculation with *P. gladioli*, the PAT content in the control group was 2.38 μg/mL, and PAT content in the ozone-treated group was 1.24 μg/mL, a decrease of 47.90% (Figure 8A). Similarly, on the 15th day after inoculation with *P. polonicum*, the PAT content in the control group was 1.51 μg/mL and 1.06 μg/mL in the ozone-treated group, a reduction of 29.80% (Figure 8B).

## 4. Discussion

In this study, the isolates causing blue mold of Lanzhou lily were isolated and purified, then identified through morphological observation and molecular biological analysis. Isolates of *T. adpressus*, *P. gladioli*, *T. calidominioluteus,* and *P. polonicum* were obtained from the naturally occurring blue mold of lily. Hahm et al. [25] identified several kinds of postharvest disease of lily bulbs during storage, and, in stabbing experiments, *Penicillium* spp. and *Fusarium* spp. were found to be the main pathogens causing bulbous rot of lily. Tang et al. [9]. investigated 26 kinds of postharvest diseases of lily in Jiangxi, of which 61.53% were attributed to fungal diseases, and they confirmed that blue mold was the main disease caused by *Penicillium* species. Feng et al. [26] found 10 kinds of lily diseases in Yuxi City, of which fungal diseases accounted for 70.00%. 

Pathogenic verification indicated that *P. gladioli* and *P. polonicum* were the main isolates leading to lily blue mold, with a disease index 77.78% and 50.00%, respectively. *P. gladioli* and *P. polonicum* infected Lanzhou lily mainly through wounds during postharvest storage. *Penicillium* spp. belong to the wound-infection type of postharvest pathogenic fungi, which mainly infect fruits and vegetables through a wound after harvest. In this experiment, we inoculated with isolates through non-destructive spraying, and no disease symptoms were observed in lily bulbs, which further indicated that the pathogens of *P. gladioli* and *P. polonicum* are wound-infection types of pathogen. During the storage and transportation of fruits and vegetables, when the tissue is mechanically damaged, a wound will provide a green channel for a pathogen to infect the host plant, and, in high-temperature and high-humidity storage conditions, which are conducive to the growth of pathogens, the disease will be more serious. Therefore, to prevent and control blue mold, on the one hand, considerable efforts should be focused on avoiding lily bruising and injuring when harvesting, storage, and transportation; on the other hand, storage conditions need to be optimized. Changing the storage conditions can achieve the purpose of slowing down the Lanzhou lily formation process.

In addition, *Penicillium* spp. can metabolize patulin under appropriate conditions. Patulin has toxicity, mutagenicity, carcinogenicity, and teratogenicity, and excessive consumption of patulin will lead to varying degrees of damage in the brain, kidney, liver, and other organs [27]. In the present study, patulin was detected in the blue-mold tissues of Lanzhou lily infected by *P. gladioli* and *P. polonicum*, with the content of 2.38 μg/mL and 1.51 μg/mL, respectively. The World Health Organization stipulates that the maximum limit [28] of patulin in fruits and their derivative products is 50 μg/kg, and the contents of patulin in lily infected with *P. gladioli* and *P. polonicum* were much higher than the maximum limit. It will do great harm to human health if the blue-mold tissue of lily is consumed. Yang et al. [29] found that patulin was also detected in the tissue of citrus blue mold caused by *P. digitatum*. Bacha et al. [30] found that the most common toxin in fruits and their derivatives is patulin produced by penicillium species. Patulin can also interact with other compounds in food and is responsible for the presence of patulin in rotten apples and apple products such as fruit juices, jams, and cider. Yu [31] reviewed patulin contamination in fruit, postharvest control methods, and the biosynthesis pathway of patulin, and suggested that patulin contamination is universal. Therefore, it is necessary to control blue mold and patulin accumulation in Lanzhou lily. Ozone, as a strong oxidant, can be naturally decomposed into oxygen, and there is no residual substance. Therefore, ozone is widely applied to control the postharvest diseases of fresh fruits and vegetables [32,33]. In this study, we found that the 2 mg/L ozone treatment significantly suppressed the development of blue mold of lily, and inhibited the accumulation of patulin. Xi et al. [18] suggested that ozone treatment effectively inhibited the development of disease caused by *Mucor hiemalis*, *Actinomucor elegans*, and *Clonostachys rosea*, and suppressed the accumulation of patulin, 15-acetyl-de-oxynivalenol, and sterigmatocystin in *Angelica sinensis*. Zapałowska et al. [34] found that ozone treatment can inhibit the growth of microorganisms, delay fruit spoilage, reduce the loss of nutritional and sensory value of fruits during storage, and ensure the extension of shelf life of fruits. Botondi et al. [35] found that low-concentration ozone can inactivate a variety of microorganisms, and gaseous ozone treatment of unprocessed fruits and vegetables has a greater impact on the final reduction in microbial load.

## 5. Conclusions

In this study, four pathogens of *T. adpressus*, *P. gladioli*, *T. calidominioluteus*, and *P. polonicum* were isolated and purified from naturally occurring blue mold of lily and identified by morphological and molecular biological techniques. The pathogenicity test showed that *P. gladioli* and *P. polonicum* had higher pathogenicity than *T. adpressus* and *T. calidominioluteus*. Therefore, *P. gladioli* and *P. polonicum* were identified as the main pathogens causing blue mold during postharvest storage of Lanzhou lily. In addition, ozone treatment with 2 mg/L significantly reduced the disease incidence and disease index of lily blue mold, and significantly inhibited the accumulation of patulin produced by *P. gladioli* and *P. polonicum* in lily. The main mechanism of disease control by ozone treatment at the transcriptomic and metabolomic levels needs to be further studied.

## Figures and Tables

**Table 1:** Primers and sequences for molecular biological identification of pathogenic pathogen.

Gene	Gene	Primer Sequence
*ITS*	*ITS1*	5′-TCCGTAGGTGAACCTGCGG-3′
	*ITS4*	5′-TCCTCCGCTTATTGATATGC-3′
*β-tubulin*	*Bt2a*	5′-GGTAACCAAATCGGTGCTGCTTTC-3′
	*Bt2b*	5′-ACCCTCAGTGTAGTGACCCTTGGC-3′

**Table 2:** Disease classification standard.

Disease Rating	Symptom
0	No disease
1	Scale disease area 0~25%
2	Scale disease area 25~50%
3	Scale disease area 50~75%
4	Scale disease area greater than 75%

## Data Availability

The data presented in this study are available in article.
